# CT-based multiple instance and ensemble learning for lymph node metastasis prediction in esophageal squamous cell carcinoma: a multicentre, retrospective study

**DOI:** 10.1186/s40644-026-01005-z

**Published:** 2026-02-14

**Authors:** Kai Qin, Jianchao Lu, Na Li, Jie Zhu, Lin Peng, Jiayu Xiang, Yi Jin, Pei Yang, Junqiang Chen, Qifeng Wang

**Affiliations:** 1 Department of Radiation Oncology, Radiation Oncology Key Laboratory of Sichuan Province, Sichuan Clinical Research Center for Cancer, Sichuan Cancer Hospital & Institute, Sichuan Cancer Center, Affiliated Cancer Hospital of University of Electronic Science and Technology of China, 55 South Renmin Ave, Fourth Section, Chengdu, Sichuan Province 610041 China; 2 Cancer Central, Suining Central Hospital, Suining, Sichuan Province China; 3 Department of Thoracic Surgery, Sichuan Clinical Research Center for Cancer, Sichuan Cancer Hospital & Institute, Sichuan Cancer Center, Affiliated Cancer Hospital of University of Electronic Science and Technology of China, Chengdu, Sichuan Province China; 4 Graduate Collaborative Training Base of Hunan Cancer Hospital, Hengyang Medical School, University of South China, Hengyang, Hunan Province China; 5 Key Laboratory of Translational Radiation Oncology, Hunan Cancer Hospital, The Affiliate Hospital of Xiangya Medical School, Central South University, No.582, Xianjiahu Road, Changsha, Hunan Province 410006 China; 6 Department of Radiation Oncology, Fujian Medical University Cancer Hospital, Fujian Cancer Hospital, No. 420, Fuma Road, Fuzhou, Fujian Province 350014 China

**Keywords:** Esophageal cancer, Radiomics, Deep learning, Lymph node metastasis

## Abstract

**Background:**

Accurately predicting lymph node metastasis (LNM) in esophageal squamous cell carcinoma (ESCC) is crucial for planning patient treatments. However, this task remains challenging, complicating treatment decision-making; this is particularly concerning for patients classified as pN0 owing to insufficient lymph node dissection (< 15 lymph nodes), as the effectiveness of postoperative adjuvant therapy (POAT) for these patients remains controversial. Therefore, we aimed to develop a CT-based predictive model to improve LNM detection in ESCC patients and identify pN0 patients who can benefit from POAT.

**Methods:**

We retrospectively enrolled 974 ESCC patients who underwent radical esophagectomy with adequate lymph node dissection (≥ 15 lymph nodes), dividing them into training (432 patients), internal validation (185 patients), and external validation cohorts (357 patients). To predict LNM, we developed a Stacking model using multiple instance and ensemble learning, leveraging the visible lymph nodes, primary tumor features, and clinical characteristics of each patient. Additionally, we separately enrolled 386 pN0 patients who underwent insufficient lymph node dissection and classified them into low-risk and high-risk groups on the basis of the optimal cut-off value from the Stacking model. Kaplan‒Meier and Cox models were used to assess the impact of POAT on patient survival.

**Results:**

The Stacking model achieved area under the curve (AUC) values of 0.883, 0.834, and 0.819 in the training, internal validation, and external validation cohorts, respectively, outperforming traditional models based separately on tumor features, clinical characteristics, or the features of the largest lymph node. pN0 patients identified by the Stacking model as high risk had worse overall survival than low-risk patients did. POAT provided survival benefits for the high-risk patients but had no significant effect on the survival of low-risk patients.

**Conclusion:**

Our Stacking model achieved excellent LNM prediction in ESCC patients and holds promise for guiding personalized treatment strategies, particularly for pN0 patients with insufficient lymph node dissection.

**Supplementary Information:**

The online version contains supplementary material available at 10.1186/s40644-026-01005-z.

## Introduction

Esophageal cancer is one of the most common malignancies of the gastrointestinal tract, ranking seventh in incidence and sixth in mortality among all cancers [1]. In China, the incidence is notably high; moreover, among all esophageal cancers, esophageal squamous cell carcinoma (ESCC) accounts for more than 90% of cases [2]. The presence of lymph node metastasis (LNM) is associated with an even worse outcome and substantially influences treatment decision-making [3]. Computed tomography (CT) is the most widely used imaging modality for diagnosing and staging esophageal cancer. In clinical practice, lymph nodes with a short axis diameter greater than 1 cm on CT are often considered malignant. However, use of this criterion achieves only 50% sensitivity and 83% specificity [4], as benign conditions such as reactive hyperplasia and inflammation can cause lymph node enlargement, whereas malignant nodes may be normal in size [5], complicating decision-making for patient treatment. Therefore, novel CT-based diagnostic approaches that can achieve better accuracy in detecting LNM in ESCC patients are urgently needed.

Currently, artificial intelligence (AI) techniques, including radiomics and deep learning, have shown great potential in disease diagnosis, patient outcome prediction, and therapeutic response assessment [6, 7]. Although previous studies have explored the feasibility of radiomics and deep learning in predicting LNM in ESCC [8, 9], they are limited by small sample sizes and a high risk of overfitting. Most existing models diagnose LNM indirectly through primary tumor imaging. To date, no study has systematically utilized imaging information from multiple lymph nodes from the same patient despite the direct diagnostic evidence provided from lymph node imaging. Moreover, traditional diagnostic algorithms have difficulties in fully utilizing the variable number of visible lymph node images in patient CT scans. To address this, we introduced an attention-guided multiple instance learning (MIL) algorithm, a semi-supervised technique that is increasingly used in AI-based medical research to handle cases with variable quantities of imaging data [10–12]. This method treats all visible lymph nodes in each patient as a “bag,” while the pathological report of the lymph node status serves as the overall label. Lymph node imaging, primary tumor imaging, and clinical characteristics could then be integrated into a comprehensive diagnostic model.

Another critical area for exploration is the use of AI models to guide postoperative adjuvant therapy (POAT) for pN0 ESCC patients who have undergone insufficient lymph node dissection. It is well established that a minimum of 15 lymph nodes should be dissected to accurately determine the N stage of the patient [13, 14]. However, owing to patient conditions and surgical limitations, some patients undergo insufficient lymph node dissection, which can lead to understaging. As per NCCN guidelines, pN0 ESCC patients are typically recommended for follow-up^14^; however, these understaged pN0 patients may have undetected LNM and could thus benefit from POAT [14, 15]. Therefore, developing AI models to identify patients inaccurately staged as pN0 who could benefit from POAT is essential.

In this study, we propose a predictive model that integrates lymph node imaging, primary tumor imaging, and clinical characteristics to increase the accuracy of LNM identification in ESCC patients. Additionally, we evaluated the clinical utility of this model in identifying pN0 patients with insufficient lymph node dissection during radical esophagectomy who may benefit from POAT.

## Methods

### Study design and patient cohorts

**Fig. 1:**
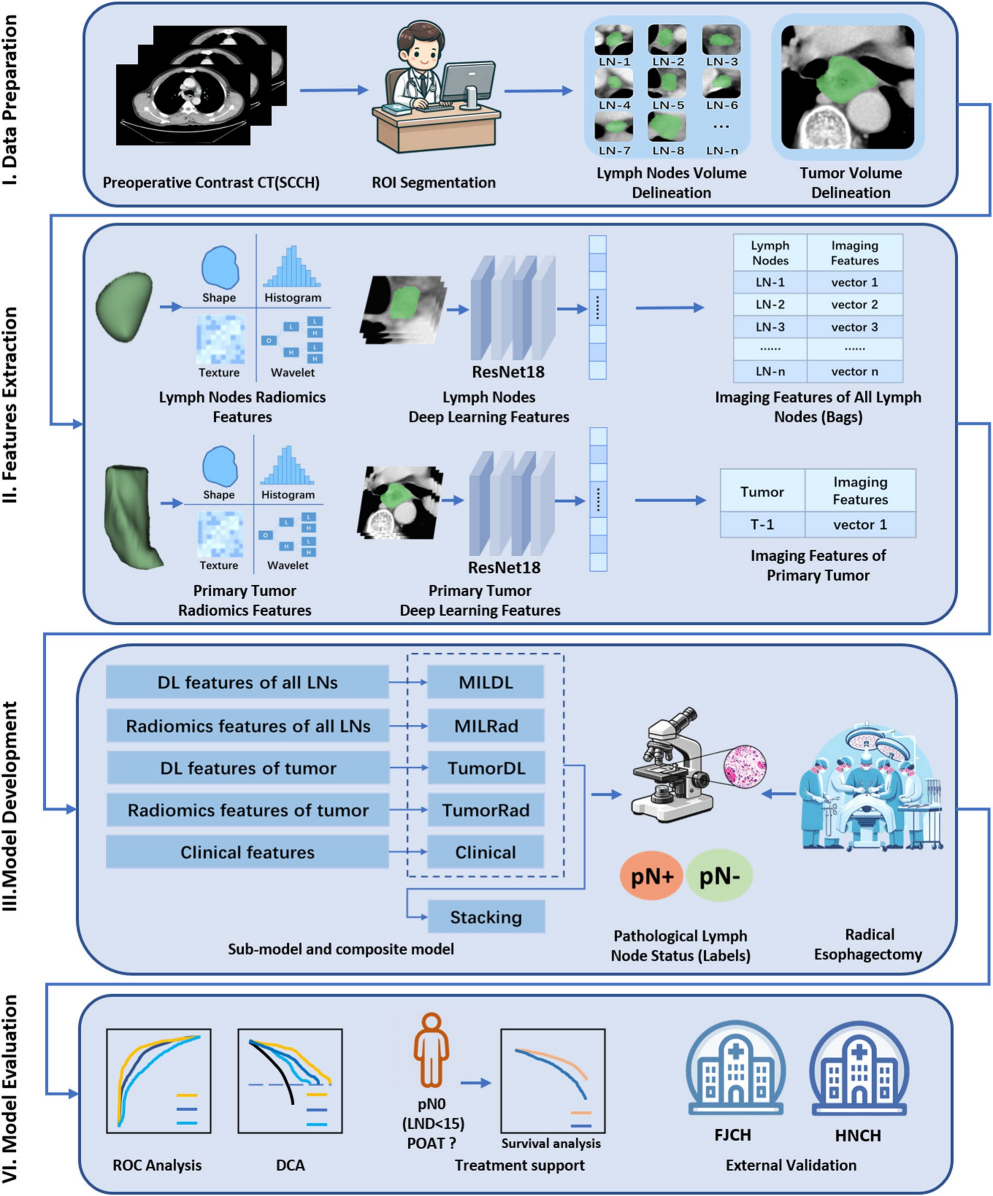
Overall study design and workflow

The study flowchart is illustrated in Fig. 1. To ensure that the pathological lymph node status was accurate throughout the study, only patients for whom at least 15 lymph nodes were dissected were included. On the basis of the inclusion and exclusion criteria, we retrospectively enrolled 974 patients with ESCC treated across three hospitals in China for model development. A total of 617 patients treated at Sichuan Cancer Hospital (SCCH) between January 1, 2011, and December 30, 2018, were randomly divided at a 7:3 ratio into a training cohort (432 patients) and an internal validation cohort (185 patients). External test cohort 1 included 185 patients from Hunan Cancer Hospital (HNCH), and external test cohort 2 included 172 patients from Fujian Cancer Hospital (FJCH), all of whom were treated between January 1, 2011, and December 30, 2018.

To evaluate the clinical benefits of the model for pN0 patients with insufficient lymph node dissection, an additional 386 patients were retrospectively enrolled from these three cancer centres. Specific details regarding patient selection can be found in the Supplementary Materials. This study was approved by the institutional review board (KYLLKS20230045) and adheres to the principles outlined in the Declaration of Helsinki.

### CT scanning and image preprocessing

All participants underwent preoperative multislice CT scanning covering the neck to the upper abdomen. During the scans, the patients were placed in the supine position and were instructed to breathe calmly and avoid swallowing to minimize motion artefacts. Contrast-enhanced images were acquired 60–65 s after intravenous contrast agent injection. The CT images were standardized via bicubic spline interpolation to a voxel size of 1 mm×1 mm×5 mm. Two experienced radiologists independently delineated the tumor boundaries and visible lymph nodes with 3D Slicer software (version 5.0.3) at the mediastinal window setting (width: 350 HU; level: 40 HU), creating regions of interest (ROIs). To assess the reliability and consistency of these delineations, all ROIs were reannotated two months later, and the corresponding intraclass correlation coefficients (ICCs) were calculated. Detailed information about the CT acquisition can be found in the Supplementary Methods.

### Radiomics feature extraction

The PyRadiomics package (https://github.com/Radiomics/PyRadiomics) was used to extract a comprehensive suite of 1,379 radiomic features [16] categorized into 7 types: (1) shape-based features, (2) first-order features, (3) grey-level dependence matrix (GLDM) features, (4) grey-level size-zone matrix (GLSZM) features, (5) neighbouring grey-tone difference matrix (NGTDM) features, (6) grey-level run-length matrix (GLRLM) features, and (7) grey-level co-occurrence matrix (GLCM) features. The detailed parameters used for the feature extraction process are provided in the Supplementary Methods.

### Radiomics feature selection and model development

The radiomic features were standardized via z-score normalization to ensure uniform scaling across different feature values, thus facilitating comparisons and further statistical analyses. We conducted Spearman correlation analysis to explore inter-feature relationships. For any pair of features with a correlation coefficient exceeding 0.9, the feature with the higher mean absolute correlation with the remaining features was removed to reduce redundancy, while the other was retained. Next, features that demonstrated high stability, that is, those with intra- and interobserver ICCs exceeding 0.75, were retained. Additional feature selection was performed with multivariate least absolute shrinkage and selection operator (LASSO) regression to further refine our model. A support vector machine (SVM) classifier was trained to construct the predictive model, employing a fivefold cross-validation approach to optimize the model configuration and hyperparameters. This SVM classifier was trained on the data from the training cohort, comprising a set of features and corresponding labels that indicated the presence of LNM. The resulting model was a trained classifier designed to predict outcomes for patients in the test cohort, outputting the probability of LNM, which ranges from 0 to 1. In this way, we developed two models: the TumorRad model, which was developed with the radiomic features of the primary tumor, and the LLNRad model, which was developed with the radiomic features of the largest lymph node.

### Deep learning feature extraction

To extract deep learning features from the lesions, we implemented a sequence of preprocessing steps. First, we identified the slice containing the largest cross-sectional area of the segmented ROI along with the slices immediately above and below it. The selected 3-layer ROI was then dilated by two voxels using a 2D slice-wise approach (in-plane expansion), and this dilated ROI was then cropped to create pseudo-3-channel images; in this way, we ensured that lesion margin information was preserved while minimizing background interference. The images were subsequently resized and adjusted to a uniform size of 224 × 224 pixels. These steps were performed for both the lymph nodes and primary tumor to extract the deep learning features. These images were processed via a pretrained ResNet18 deep learning model, which is widely used in many medical studies [17, 18]. The features were specifically extracted from the final pooling layer of the ResNet18 model, yielding a total of 512 features. This method capitalizes on the architectural advantages of ResNet18, enabling the distillation of complex spatial relationships within and around the lesions into a comprehensive feature set. Consequently, this enhances the ability of the model to characterize the morphology of the lesion accurately based on these detailed features. These ResNet18 features were used to train independent deep learning sub-models. Since a late fusion strategy was adopted where sub-models were trained independently, the ResNet18 features were not z-score normalized prior to modeling. Additional details on the deep learning feature extraction process are available in the Supplementary Methods.

### MIL model development

To fully harness the potential of imaging data from multiple lymph nodes, we developed a framework that utilizes attention-guided MIL techniques. This innovative framework processes sets of lymph node features—derived from both deep learning and radiomics—and assesses the likelihood of the patient having LNM. In this context, a “bag” represents an individual patient, and each “instance” corresponds to a feature of each lymph node. Importantly, labels are assigned to the entire bag rather than to individual instances. Our MIL network contains the following components:

Embedding layer: The MIL process begins with a sequential network consisting of fully connected layers, rectified linear unit (ReLU) activation functions, and a Dropout layer, the last of which reduces dimensionality and weights the features according to the imaging characteristics of the lymph node. This layer can extract 32-dimensional embedded representations that increase the effectiveness of the subsequent attention mechanism.

Attention layer: A specialized attention network then compresses the 32-dimensional features into a single dimension, assigning attention scores to each instance. A tanh activation function helps the model capture complex interrelationships among instances, whereas a Softmax function normalizes attention scores into weights. This allows the model to focus autonomously on the most diagnostically significant lymph nodes, enhancing the diagnostic accuracy of the output.

Classification layer: This layer aggregates the weighted feature representations from the attention mechanism and outputs binary predictions that indicate the presence or absence of LNM.

During the training process, the parameters of the MIL network were iteratively updated through backpropagation, and the focal loss function was employed. L1 regularization was used to mitigate overfitting and facilitate feature selection. The Adam optimizer was used to update the parameters with a learning rate set at 1 × 10^− 4^ and a weight decay of 1 × 10^− 6^. The batch size was set to 32.

Two MIL models, MILRad and MILDL, were developed to analyze comprehensive sets of radiomic and deep learning features, respectively, extracted from the visible lymph nodes. Further details about the MIL models are available in the Supplementary Methods.

### Clinical model development

The clinical characteristics listed in Table 1 were used to construct the clinical model. These features were standardized via z-score normalization and refined with LASSO regression, after which the resulting features were used to train an SVM classifier for prediction.

**Table 1:** Baseline characteristics of study cohorts

Characteristics	Entire (*n* = 974)	Training Cohort (*n* = 432)	Internal Validation Cohort (*n* = 185)	External Validation Cohort (*n* = 357)	*p* value^a^
Age, n(%)					0.006
<65	627(64.37)	285(65.97)	133(71.89)	209(58.54)	
≥65	347(35.63)	147(34.03)	52(28.11)	148(41.46)	
Sex, n(%)					0.540
Male	816(83.78)	359(83.10)	160(86.49)	297(83.19)	
Female	158(16.22)	73(16.90)	25(13.51)	60(16.81)	
Primary site, n(%)					0.042
Upper	246(25.26)	122(28.24)	45(24.32)	79(22.13)	
Middle	545(55.95)	229(53.01)	96(51.89)	220(61.62)	
Lower	183(18.79)	81(18.75)	44(23.78)	58(16.25)	
T stage, n(%)					0.084
T1	75(7.70)	31(7.18)	14(7.57)	30(8.40)	
T2	184(18.89)	86(19.91)	31(16.76)	67(18.77)	
T3	616(63.24)	281(65.05)	125(67.57)	210(58.82)	
T4	99(10.16)	34(7.87)	15(8.11)	50(14.01)	
Tumor length (cm)	4.19 ± 1.84	4.18 ± 1.89	4.26 ± 1.83	4.18 ± 1.78	0.973
Lymph node metastasis, n(%)					0.377
No	371(38.09)	159(36.81)	66(35.68)	146(40.90)	
Yes	603(61.91)	273(63.19)	119(64.32)	211(59.10)	

^a^p value was calculated by Chi-square test or Kruskal-Wallis test

### Composite model development

We adopted decision-level fusion, also known as late fusion, to construct a composite model by integrating the output probabilities from the multiple predictive models described above. We utilized a stacking strategy in which the probabilities from the five base models are combined to enhance the prediction accuracy. SVM was chosen for further analysis. The optimal hyperparameters for the SVM classifier were determined through a fivefold cross-validation process on the training cohort. The refined Stacking model was subsequently assessed in the internal and external validation cohorts.

### Statistics analysis

Categorical variables were compared with the chi-square test or Fisher’s exact test, whereas continuous variables were analysed with the Mann‒Whitney U test or Kruskal-Wallis test. Model performance was evaluated with the AUC. Additional performance metrics, including accuracy, sensitivity, specificity, positive predictive value (PPV), and negative predictive value (NPV), were calculated according to the cut-off determined by the maximum Youden index in the training set. Kaplan‒Meier survival curves and log-rank tests were used to assess patient stratification by the proposed feature signature. A p value less than 0.05 was considered to indicate statistical significance. Statistical analyses were performed with R (version 4.3.2) and the scikit-learn package (version 1.3.1) in Python 3.11.3.

## Results

### Study design and baseline information

The baseline characteristics of the patients in the training cohort, internal validation cohort, and external validation cohort are detailed in Table 1. A total of 974 patients with ESCC met the inclusion and exclusion criteria and were divided into a training cohort (432 patients) internal validation cohort (185 patients), and external validation cohort (357 patients; Supplemental Figure S1). A total of 8,265 visible lymph nodes were annotated, an average of 8.73 lymph nodes per patient. Among the study patients, 64.37% (*n* = 637) were younger than 65, and 83.78% (*n* = 816) were male. The primary tumor site was the upper segment in 25.26% (*n* = 246), the middle segment in 55.95% (*n* = 545), and the lower segment of the esophagus in 18.79% (*n* = 183) of the patients. With respect to tumor stage, 7.70% (*n* = 75) of the patients had T1 stage, 18.89% (*n* = 184) had T2 stage, 63.24% (*n* = 616) had T3 stage, and 10.16% (*n* = 99) had T4 stage cancer. The tumor differentiation status was classified as well differentiated in 14.48% (*n* = 141), moderately differentiated in 82.24% (*n* = 801), and poorly differentiated or undifferentiated in 3.29% (*n* = 32) of the patients. Lymph node metastasis was diagnosed in 61.91% (*n* = 603) of the patients. Statistically significant differences in age were observed among the three study cohorts (training, internal validation, and external validation; *p* = 0.006). Similarly, differentiation status showed significant heterogeneity among the cohorts (*p* < 0.001). In contrast, no significant differences were found in the distributions of sex (*p* = 0.540) or lymph node metastasis (*p* = 0.377).

The baseline characteristics of the pN0 patients who underwent insufficient lymph node dissection are provided in Supplementary Table S1.

### Model development and performance

**Fig. 2:**
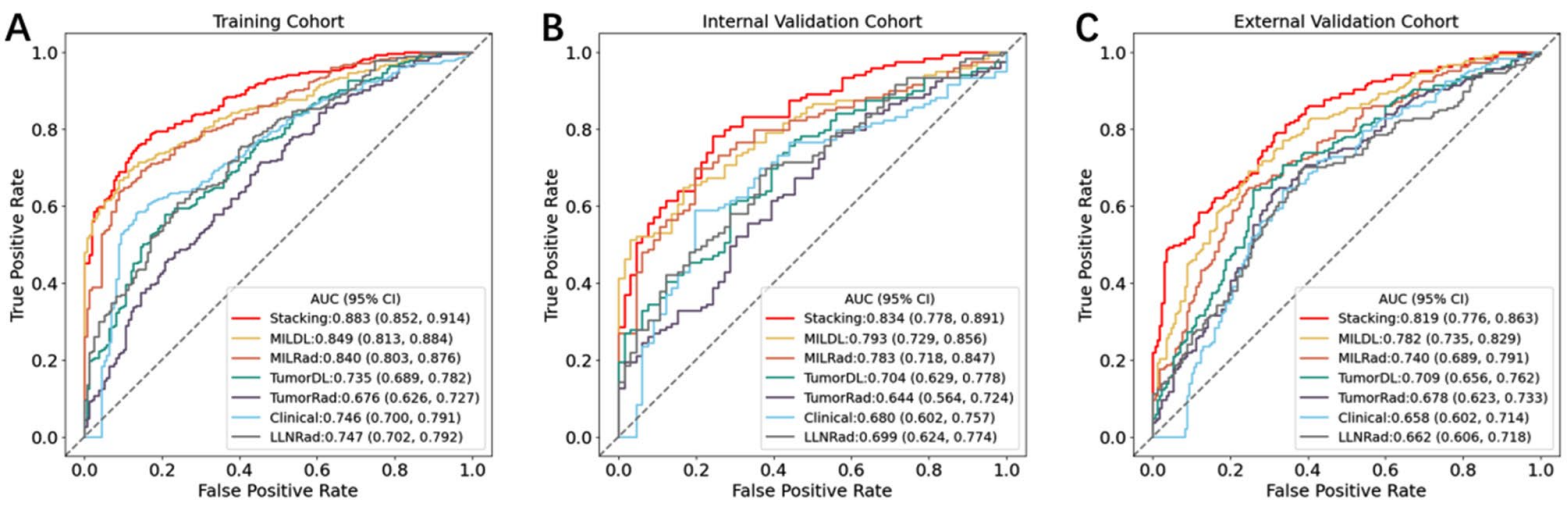
Performance for LNM prediction. The receiver operating characteristic (ROC) curves of models in the training cohort (**A**), internal validation cohort (**B**), and external validation cohort (**C**)

The diagnostic performance of the predictive models is summarized in Table 2; Fig. 2. Among all the models evaluated, the Stacking model achieved the highest performance in all cohorts, with AUCs of 0.883 in the training cohort, 0.834 in the internal validation cohort, and 0.819 in the external validation cohort. The Stacking model significantly outperformed both the clinical model and the separate primary tumor and largest lymph node feature models in all cohorts. Additionally, the two MIL models, MILDL and MILRad, demonstrated satisfactory AUCs, surpassing those of the LLNRad model in all cohorts.

**Table 2:** Performances of the predictive models in the training, internal validation, and external validation cohort

Model and metric	AUC^a^	Accuracy (%)	Specificity (%)	Sensitivity (%)	PPV (%)	NPV (%)
Training Cohort						
Stacking	0.883(0.852–0.914)	0.803	0.855	0.762	0.900	0.677
MILDL	0.849(0.813–0.884)	0.766	0.887	0.689	0.913	0.624
MILRad	0.84(0.803–0.876)	0.759	0.899	0.648	0.917	0.598
TumorDL	0.735(0.689–0.782)	0.699	0.774	0.531	0.801	0.49
TumorRad	0.676(0.626–0.727)	0.676	0.547	0.711	0.729	0.524
Clinical	0.746(0.700-0.791)	0.727	0.899	0.626	0.914	0.584
LLNRad	0.747(0.702–0.792)	0.711	0.761	0.608	0.814	0.531
Internal Validation Cohort						
Stacking	0.834(0.778–0.891)	0.773	0.758	0.773	0.852	0.649
MILDL	0.793(0.729–0.856)	0.724	0.833	0.647	0.875	0.567
MILRad	0.783(0.718–0.847)	0.741	0.803	0.697	0.865	0.596
TumorDL	0.704(0.629–0.778)	0.692	0.712	0.555	0.776	0.470
TumorRad	0.644(0.564–0.724)	0.659	0.47	0.756	0.72	0.517
Clinical	0.734(0.662–0.805)	0.719	0.803	0.639	0.854	0.552
LLNRad	0.699(0.624–0.774)	0.686	0.636	0.681	0.771	0.525
External Validation Cohort						
Stacking	0.819(0.776–0.863)	0.739	0.682	0.791	0.733	0.748
MILDL	0.782(0.735–0.829)	0.717	0.718	0.717	0.736	0.697
MILRad	0.74(0.689–0.791)	0.695	0.765	0.631	0.747	0.653
TumorDL	0.709(0.656–0.762)	0.683	0.724	0.647	0.72	0.651
TumorRad	0.678(0.623–0.733)	0.658	0.671	0.647	0.684	0.633
Clinical	0.658(0.602–0.714)	0.653	0.665	0.642	0.678	0.628
LLNRad	0.662(0.606–0.718)	0.655	0.606	0.701	0.662	0.648

Abbreviations: AUC, area under the curve; PPV, positive predictive value; NPV, negative predictive value. ^a^Data in parentheses are 95% CIs

The optimal cut-off values of the predictive models, determined on the basis of the maximum Youden index calculated in the training set, were 0.530 for the Stacking model, 0.550 for MILDL, 0.500 for MILRad, 0.484 for TumorDL, 0.526 for TumorRad, 0.400 for the clinical model, and 0.322 for LLNRad. According to the optimal cut-off values, the Stacking model exhibited the highest accuracy across both validation cohorts.

**Fig. 3:**
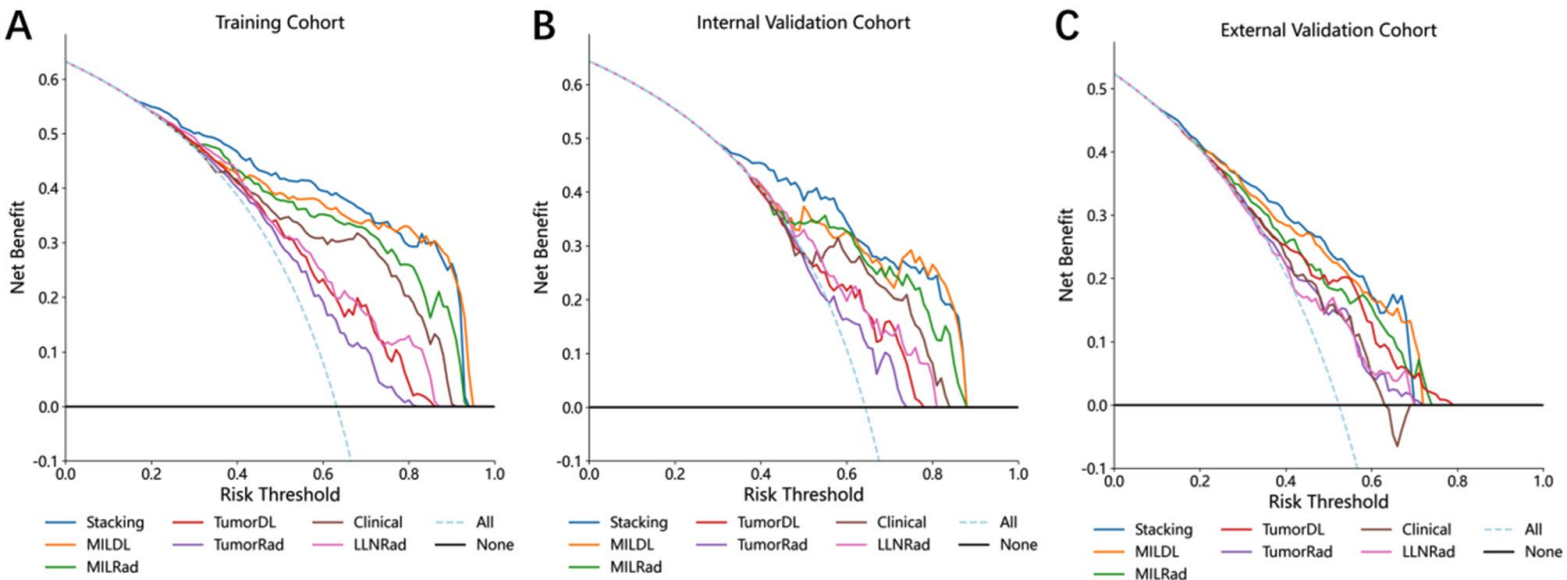
Decision curve analysis for each model in the training cohort (**A**), internal validation cohort (**B**), external validation cohort (**C**)

Decision curve analysis (DCA) further confirmed the superiority of the Stacking model, as it provided greater net benefits than the other models, as shown in Fig. 3.

### Decision-making support for POAT

**Fig. 4:**
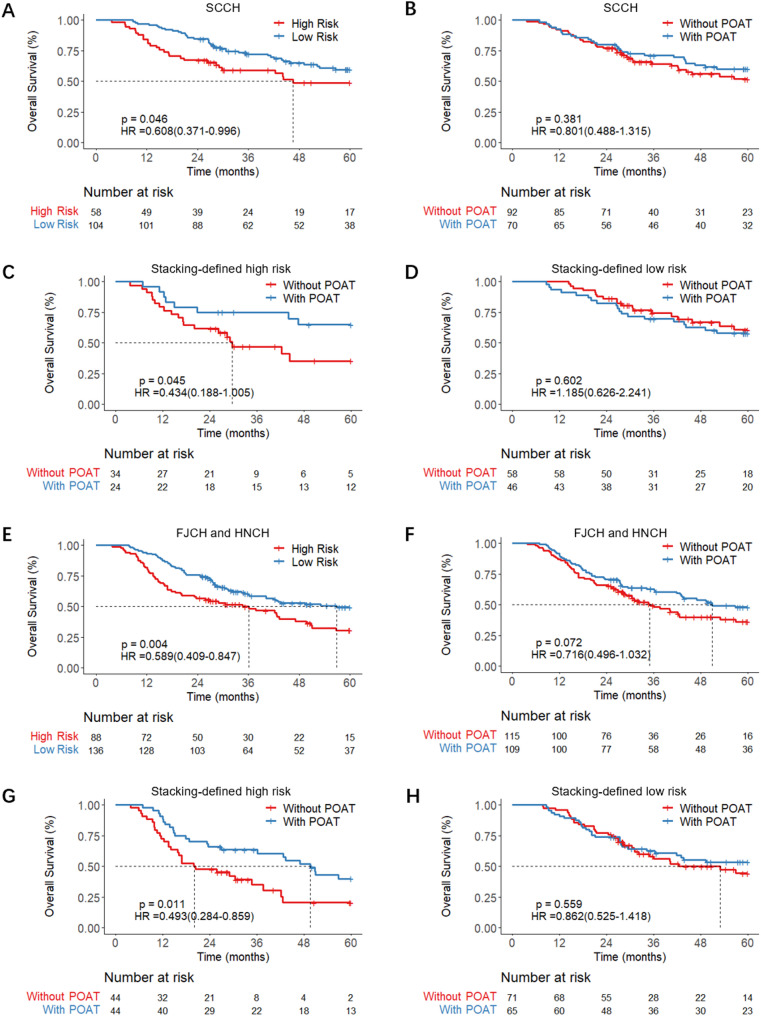
Prognosis of patients with pN0 status and insufficient lymph node dissection. (**A**-**D**) Results from SCCH; (**E**-**H**) Results from FJCH and HNCH. Survival comparisons between: (**A**, **E**) High-risk versus low-risk group; (**B**, **D**) With adjuvant therapy versus without adjuvant therapy; (**C**, **G**) With adjuvant therapy versus without adjuvant therapy in the high-risk group; (**D**, **H**) With adjuvant therapy versus without adjuvant therapy in the low-risk group

In the cohort of pN0 patients with insufficient lymph node dissection, the Stacking model was used to classify patients into low-risk and high-risk groups. Survival analysis demonstrated that a cut-off Stacking score of 0.53 could significantly stratify patient outcomes at the SCCH (HR = 0.608 [0.371–0.996], *p* = 0.046) and jointly at the FJCH and HNCH (HR = 0.589 [0.409–0.847], *p* = 0.004), as illustrated in Fig. 4A and E. However, overall survival analyses suggested that POAT did not improve survival outcomes across the three hospitals, as the corresponding survival curves for the low-risk and high-risk groups were not significantly different, as shown in Fig. 4B and F. Notably, within the high-risk group, patients who received POAT at SCCH had significantly improved outcomes compared with those who did not, demonstrating a higher five-year overall survival (OS) rate (HR = 0.434 [0.188–1.005], *p* = 0.045), as shown in Fig. 4C. Similar findings were observed at FJCH and HNCH, where POAT significantly improved high-risk patient outcomes (HR = 0.493 [0.284–0.859], *p* = 0.011), as shown in Fig. 4G. Conversely, in the low-risk group, the five-year OS rate between SCCH patients who did and did not receive POAT was comparable, indicating that the therapy had no significant benefit (HR = 1.185 [0.626–2.241], *p* = 0.602); this pattern was also consistent at FJCH and HNCH (HR = 0.862 [0.525–1.418], *p* = 0.559), as shown in Fig. 4D and H. Cox regression models were employed to assess the influence of various clinical and demographic factors on the survival outcomes of high-risk pN0 patients with insufficient lymph node dissection. Multivariable Cox regression analysis demonstrated that POAT significantly reduced the risk of mortality for patients at SCCH (HR = 0.45 [0.20–0.99], *p* = 0.047). Similar results were observed for the patients at FJCH and HNCH, where POAT was associated with significant survival improvements (HR = 0.51 [0.29–0.88], *p* = 0.015). (Supplementary Materials).

## Discussion

In this retrospective, multicentre study, we developed and validated a composite model that predicts LNM in ESCC with MIL and ensemble learning techniques. Our model effectively leveraged CT imaging data from both the primary tumor and the lymph nodes of each patient and achieved highly accurate predictions of LNM. The Stacking model exhibited favourable discriminative ability, with AUCs of 0.882 in the training cohort, 0.834 in the internal validation cohort, and 0.817 in the external validation cohort. These results significantly exceeded those of models based solely on the primary tumor features, the features of the largest lymph node, or clinical characteristics. Additionally, when the model was input data from pN0 patients who underwent insufficient lymph node dissection, patients identified as high risk for LNM by the model had poorer survival outcomes. More importantly, these high-risk patients could benefit from POAT. Thus, our model has potential for enhancing medical decision-making and personalizing treatment strategies.

To our knowledge, this study is the first to comprehensively employ imaging information from both the primary tumor and lymph nodes for diagnosing LNM in ESCC. To fully exploit the potential of the features of primary lesions and lymph nodes and of clinical data, we adopted a late fusion strategy [19], integrating submodels into a composite model to optimize the precision of LNM predictions. This composite model achieved the highest AUC, outperforming models based on the features of the primary lesion, the features of the largest lymph node, or clinical data alone.

CT remains the primary imaging modality for ESCC [20]. In clinical practice, the assessment of lymph node status heavily relies on clinicians’ subjective interpretations of CT images. Unfortunately, the diagnostic accuracy of these assessments is suboptimal [21], directly impacting patient outcomes and treatment strategies. Although positron emission tomography (PET), which leverages tissue metabolic characteristics to image the body, allows greater diagnostic accuracy than CT, its application is limited because of concerns regarding radioactivity and high costs [22]. Endoscopic ultrasound with fine-needle aspiration (EUS-FNA) has also demonstrated greater accuracy than CT in evaluating cN staging [23]; however, it is invasive and similarly costly. Therefore, there is a need for accurate, noninvasive models to diagnose LNM effectively.

Radiomics and deep learning, which extract high-dimensional features from radiological images, offer a promising approach for more accurately assessing the lymph node status of patients with ESCC. Some studies have utilized radiomic models to assess LNM in cancer patients [24–26]. However, most existing studies indirectly predicted LNM on the basis of features from the primary tumor alone. Other studies have shown that the radiomic features of lymph nodes have potential in determining nodal malignancy [27–29]. Thus, in ESCC, numerous visible lymph nodes on a CT scan can provide direct evidence of a patient’s lymph node status. However, diagnoses based on a single lesion, such as the largest lymph node, do not fully utilize these images and limit further improvements to model accuracy. To fully leverage the imaging information from lymph nodes, we employed an MIL approach, which is increasingly utilized in medical AI [30–32]. This method treats the numerous visible lymph nodes on a patient’s CT scan as a collective “bag,” whose overall label is the pathological lymph node status. We further integrated an attention mechanism [33], enhancing our model’s ability to focus on critical lymph node features during diagnosis. This approach assigns attention weights to lymph node instances, thereby improving the interpretability and performance of the model. In our study, each patient had an average of 8.37 annotated lymph nodes. The MIL algorithm allows us to analyse all visible lymph nodes comprehensively rather than focusing solely on a single or even the largest lymph node, thereby enabling a more precise evaluation of a patient’s lymph node status.

Integrating radiomic signatures into clinical workflows to optimize treatment decisions represents a crucial benchmark for demonstrating their clinical utility. Unlike studies limited to model construction and efficiency evaluation, in this study, we explored potential application scenarios for the proposed composite model. For patients with ESCC undergoing radical esophagectomy, although POAT can eradicate residual micrometastatic disease for ESCC patients undergoing radical esophagectomy, it can result in significant side effects, making proper patient selection crucial [34, 35]. The applicability of POAT for pN0 patients with insufficient lymph node dissection remains controversial, as not all such patients would benefit from this treatment. According to current clinical guidelines, close monitoring is recommended for pN0 patients [14]. Selecting appropriate patients for POAT continues to pose a significant challenge. Our results revealed that patients who received POAT on the basis of clinicians’ judgement had similar OS to those who did not receive POAT. When we input the CT images of these patients to our Stacking model, the outputs allowed us to categorize them into high risk and low risk for LNM on the basis of established diagnostic thresholds. Prognostically, the high-risk group exhibited significantly poorer outcomes, indicating that some patients, despite being diagnosed with pN0 disease due to insufficient lymph node dissection, might still harbour undetected lymph node metastases. Comparative analyses between the high- and low-risk groups revealed that the former, but not the latter, could derive survival benefits from POAT. Thus, our AI model could aid in identifying potentially undetected LNM among patients classified as pN0 due to insufficient lymph node dissection and guide the development of appropriate postoperative treatment strategies.

Despite the encouraging findings, this study has several limitations. First, as a multicentre retrospective study, our findings must be further verified through prospective research. Second, our analysis was limited to patients with squamous cell carcinoma, which is the most prevalent form of esophageal cancer in China; future investigations should assess the model’s applicability to patients with adenocarcinoma. Third, the lymph node ROIs were manually delineated by clinicians, a process that is inherently time-consuming. Future efforts will explore the potential of automated segmentation for delineating the ROIs. Finally, we did not guide postoperative treatment on the basis of postoperative contrast-enhanced CT, as it is not routinely performed during the perioperative period after esophageal cancer surgery in China.

In summary, we developed a model that effectively employs MIL and ensemble learning to comprehensively utilize imaging data from lymph nodes and primary tumors as well as clinical features, achieving robust accuracy in noninvasively predicting LNM and outperforming previously proposed models. Our Stacking model shows promise for guiding adjuvant treatment decision-making in patients with ESCC who are diagnosed with pN0 disease due to inadequate lymph node dissection. Prospective trials should be conducted to further refine and validate the clinical utility of our proposed model.

## Supplementary Information

Below is the link to the electronic supplementary material.

Supplementary Material 1

Supplementary Material 2

## Data Availability

All the data are available from corresponding authors (littlecancer@163.com) upon reasonable request.
