# Supplementary material for: CT-based multiple instance and ensemble learning for lymph node metastasis prediction in esophageal squamous cell carcinoma: a multicentre, retrospective study
Source: Cancer Imaging. 2026 Feb 14;26:40. doi: 10.1186/s40644-026-01005-z (PMC13011690; doi:10.1186/s40644-026-01005-z)
Supplement: Supplementary file 1 — Supplementary Material 1 [file 40644_2026_1005_MOESM1_ESM.docx]

**Supplementary materials**

**CT-based multiple instance and ensemble learning for lymph node metastasis prediction in esophageal squamous cell carcinoma: a multicentre, retrospective study**

**Supplementary Methods**

**1.CT acquisition**

Sichuan cancer hospital: CT scancer: Philips iCT 256; Philips Medical Systems, Best, Netherlands; Parameters: tube voltage of 120 kVp, tube current of 100–370 mAs, slice thickness = 1.0 mm. Contrast medium: The patients were injected intravenously with 1.5 ml/kg contrast medium (Visipaque 320, GE Healthcare) into the antecubital vein at a rate of 2.5 ml/s via a power injector (Missouri XD2001, Ulrich GmbH&Co, Buchbrunnenweg, Ulm, Germany).

Hunan cancer hospital：multidetector row CT system (Discovery CT750HD, GE Healthcare); Parameters: tube voltage of 120 kVp, tube current of 250–400 mAs, and slice thickness of 0.625 mm. Contrast medium: The patients were injected intravenously with 1.5 ml/kg contrast medium (Ultravist 370, Bayer Schering Pharma) into the antecubital vein at a rate of 3.0 ml/s via an automated power injector.

Fujian cancer hospital：multidetector row CT system (Discovery CT750HD, GE Healthcare); Parameters: tube voltage of 120 kVp, tube current of 250–400 mAs, and slice thickness of 0.625 mm. Contrast medium: The patients were injected intravenously with 1.5 ml/kg contrast medium (Ultravist 370, Bayer Schering Pharma) into the antecubital vein at a rate of 3.0 ml/s via an automated power injector.

**2.Radiomics feature extraction**

This study utilized the majority of default parameter settings provided by the PyRadiomics package. A range of image filters and feature classes were applied to capture both intensity-based and texture-based characteristics.

First, the images underwent preprocessing, where voxel values were normalized using a normalization scale of 1000, ensuring comparability across different scanners. Additionally, the images were resampled to an isotropic spacing of 1 mm using the nearest-neighbor interpolator. Padding was applied to account for the large sigma values in the Laplacian of Gaussian (LoG) filter, with a pad distance set to 10. Mask validation was enabled to ensure proper mask generation.

Multiple image filters were applied, including Original, Laplacian of Gaussian (LoG) with sigma values of 1.0, 2.0, and 3.0, Wavelet, 3D Local Binary Patterns (LBP3D), Exponential, Square, Square Root, Logarithm, and Gradient filters, highlighting different aspects of the imaging data by capturing fine details and larger scale structures.

The radiomic features extracted were divided into several categories: shape-based features, first-order statistics, and second-order texture features, including gray-level co-occurrence matrix (GLCM), gray-level run-length matrix (GLRLM), gray-level size zone matrix (GLSZM), gray-level dependence matrix (GLDM), and neighboring gray-tone difference matrix (NGTDM). For the GLCM, all available features were extracted except for SumAverage.

For image discretization, the bin width was set to 25 to ensure an appropriate number of bins in the range of 16–128, based on the intensity range of the dataset. Additionally, a voxel array shift of 1000 was applied to account for negative Hounsfield units in the CT images, ensuring non-negative values during the feature extraction process.

**Deep learning feature extraction**

The data pre-processing protocol was structured into eight steps to ensure optimal preparation for deep learning. These steps include: (1) Resampling each voxel in the original images to a spatial resolution of 1mm×1mm×5mm; (2) Identifying the largest cross-sectional layer of the segmented region of interest (ROI) along with its adjacent upper and lower slices. This 3-layer ROI was then dilated by two voxels, and the dilated ROI was subsequently cropped to generate pseudo-3-channel images of dimensions; (3) Rotating these pseudo-3-channel images randomly between -180° and 180° around their center; (4) Randomly shifting the lesion location within a range of -3 to +3 pixels; (5) Randomly cropping and resizing the image stacks to 224×224; (6) Randomly sharpening the image stacks; (7) Randomly blurring the image stacks; (8) Normalizing the images by subtracting the mean and dividing by the variance.

The input of the feature extractor was a tensor of size 3×224×224, processed through the ResNet-18 architecture, which includes 8 residual blocks. The pretrained ResNet-18 based on ImageNet dataset was utilized, which offers improved parameter initialization tailored for medical imaging tasks. The feature extractor outputs a map that is classified by a dual-layered network: the first layer has 512 output nodes, and the second contains two nodes. These nodes apply a softmax function to output two probabilities: the likelihood of metastasis and non-metastasis, which sum to one. This architecture ensures precise predictions crucial for clinical applications.

**Figure S1: The flow chart of patient inclusion.**

Patient cohorts for model development: the inclusion criteria were as follows: (1) Confirmed ESCC diagnosis via surgical pathology; (2) complete pre-surgical contrast-enhanced CT image data and clinical data; (3) post-radical esophagectomy pathological lymph node status confirmation, with a minimum of 15 lymph nodes dissected; (4) absence of any neoadjuvant therapy prior to the surgery.


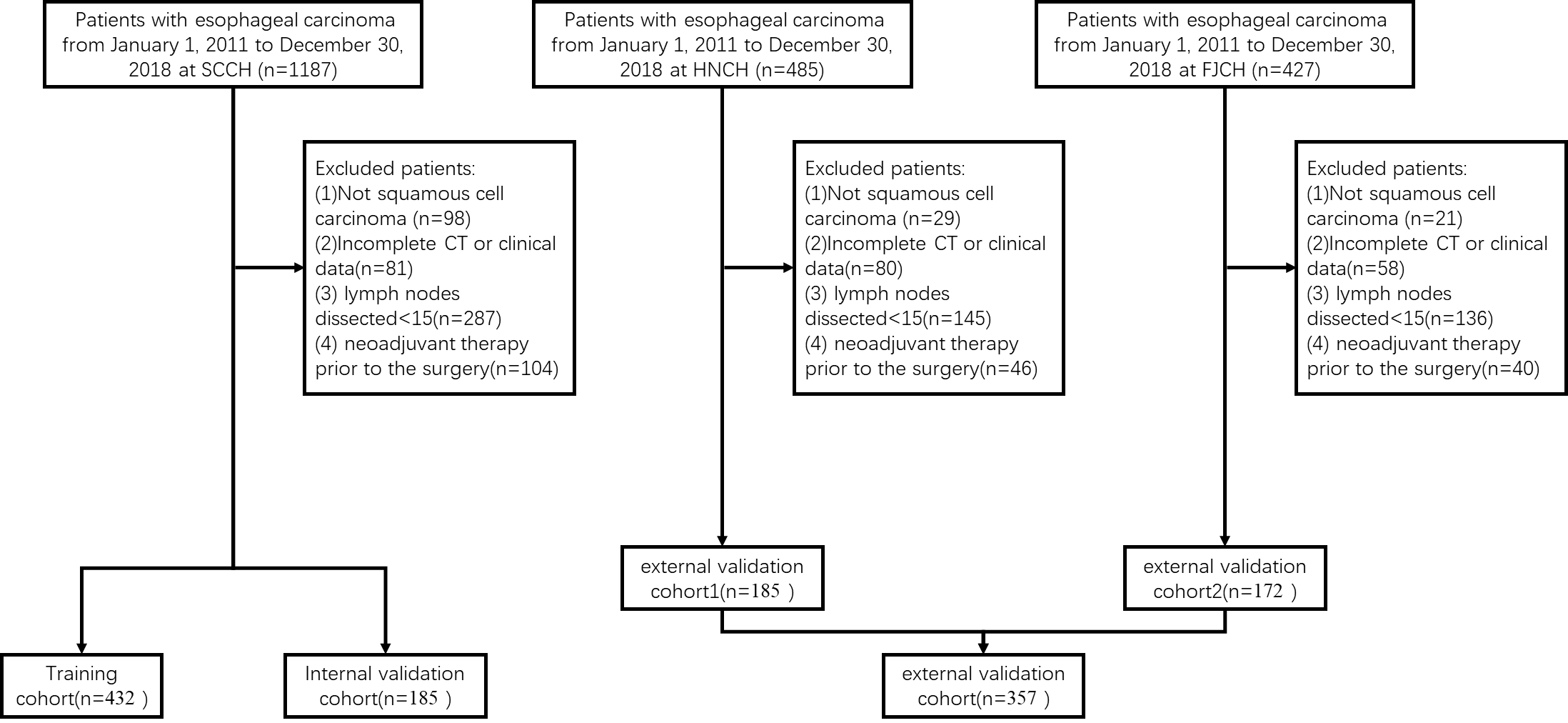


Moreover, to assess the clinical utility of the Stacking model in guiding POAT for patients with pN0 status who underwent insufficient lymph node dissection, a cohort from SCCH, FJCH and HNCH was assembled. Inclusion criteria were as follows: (1) post-radical esophagectomy pathological lymph node status confirmation, with a maximum of 14 lymph nodes dissected and postoperative pathology confirmed pN0 status; (2) confirmed ESCC diagnosis via surgical pathology; (3) complete pre-surgical contrast-enhanced CT image data and clinical data; (4) absence of any neoadjuvant therapy prior to the surgery; (5) availability of follow-up data for both disease-free survival (DFS) and overall survival (OS).


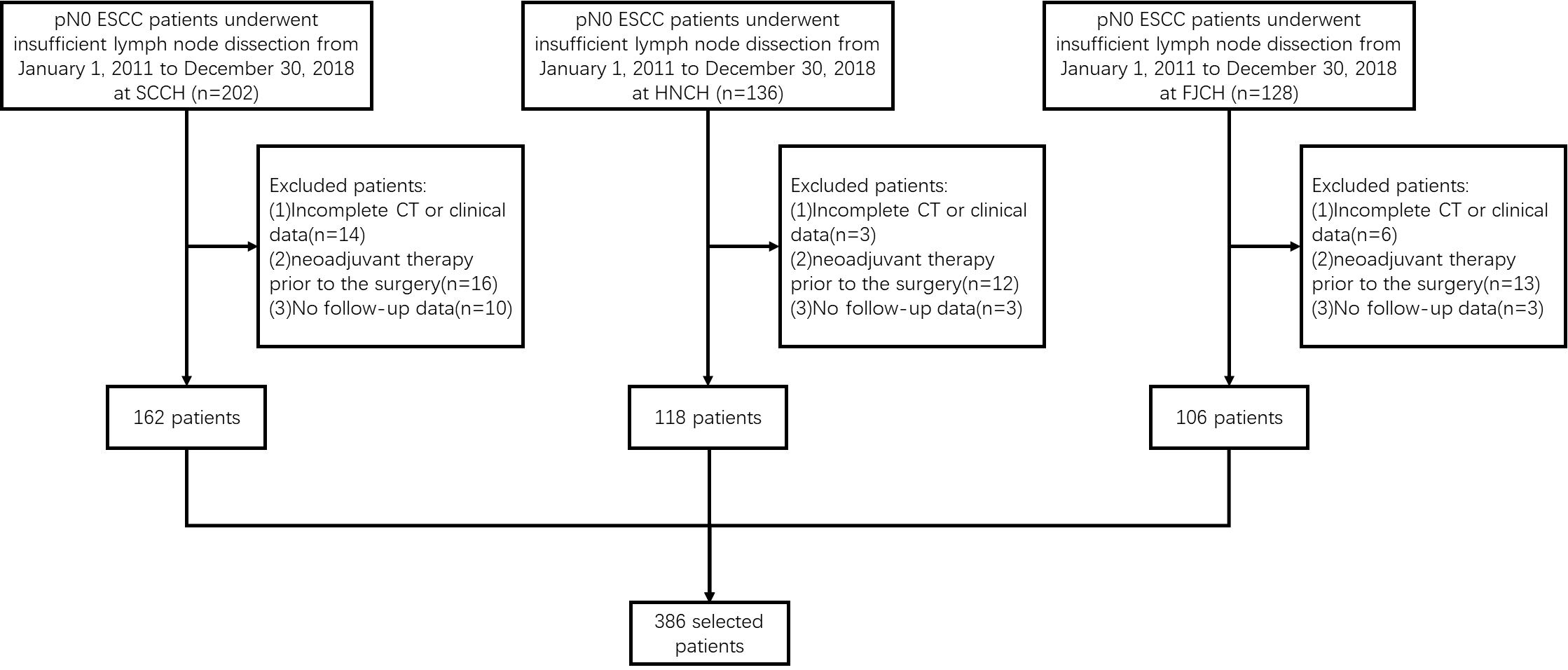


**Table S1:** **Baseline characteristics of pN0 study cohorts.**

| **Characteristics** | **SCCH**  **(n=162)** | **HNCH**  **(n=118)** | **FJCH**  **(n=106)** |
| --- | --- | --- | --- |
| Age, n(%) |  |  |  |
| ＜65 | 96(59.26) | 78(66.10) | 67(63.21) |
| ≥65 | 66 (40.74) | 40(33.90) | 39(36.79) |
| Sex, n(%) |  |  |  |
| Male | 125(77.16) | 97(82.20) | 89(83.96) |
| Female | 37(22.84) | 21(17.80) | 17(16.04) |
| Primary site, n(%) |  |  |  |
| Upper | 46(28.40) | 30(25.42) | 23(21.70) |
| Middle | 86(53.09) | 62(52.54) | 54(50.94) |
| Lower | 30(18.52) | 24(20.34) | 29(27.36) |
| T stage, n(%) |  |  |  |
| T1 | 17(10.49) | 7(5.93) | 8(7.55) |
| T2 | 49(30.24) | 27(22.88) | 22(20.75) |
| T3 | 86(53.09) | 70(59.32) | 65(61.32) |
| T4 | 10(6.17) | 14(11.86) | 11(10.38) |
| Tumor length (cm) | 3.73±1.99 | 3.96±2.02 | 3.92±1.87 |

High-risk pN0 with insufficient lymph node dissection at SCCH：

Univariate Cox Regression Analysis


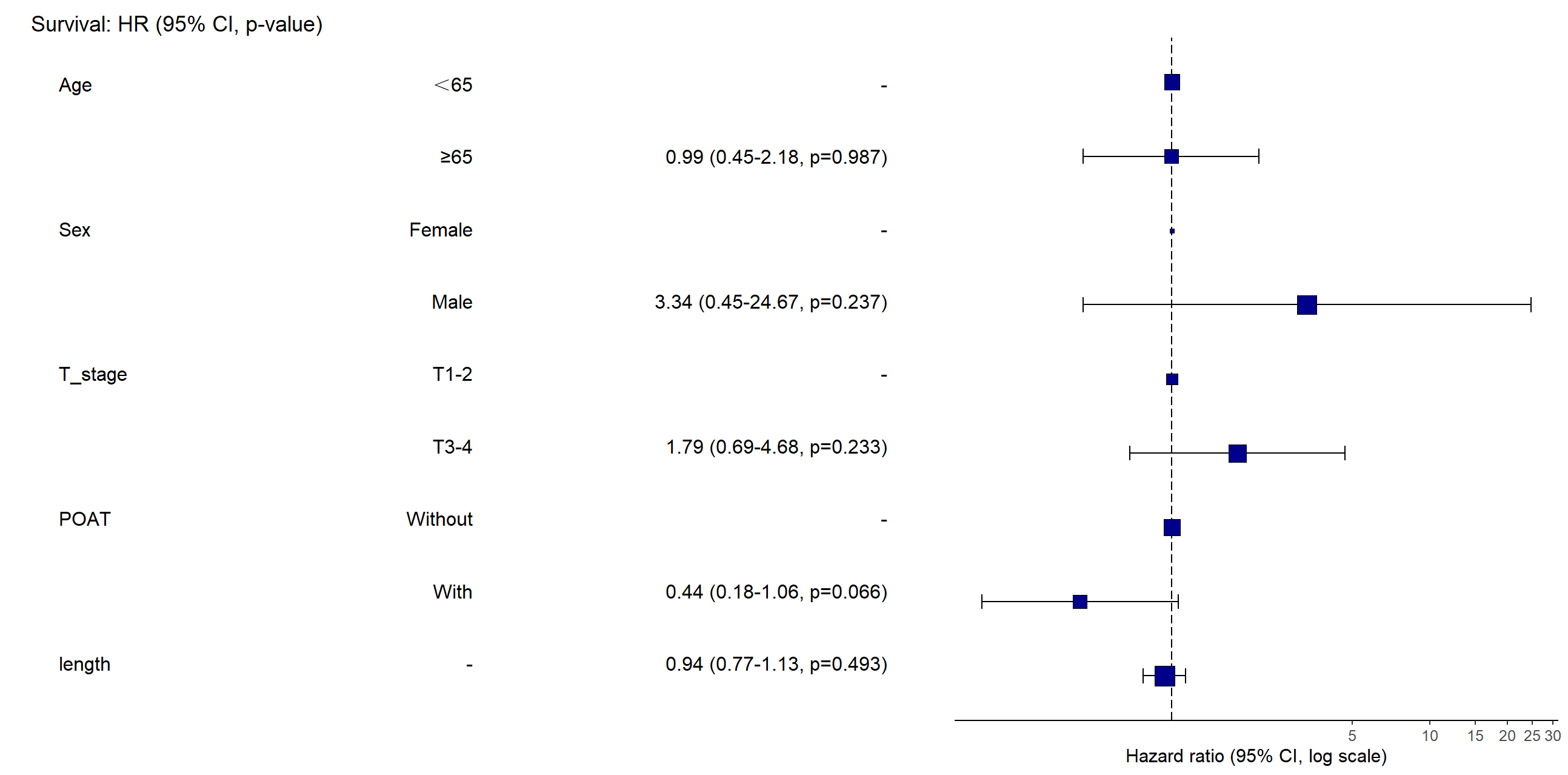


Note: Tumor length was modeled as a continuous variable (no cutoff applied). The hazard ratio corresponds to the risk associated with each 1-cm increase.

Multivariate Cox Regression Analysis


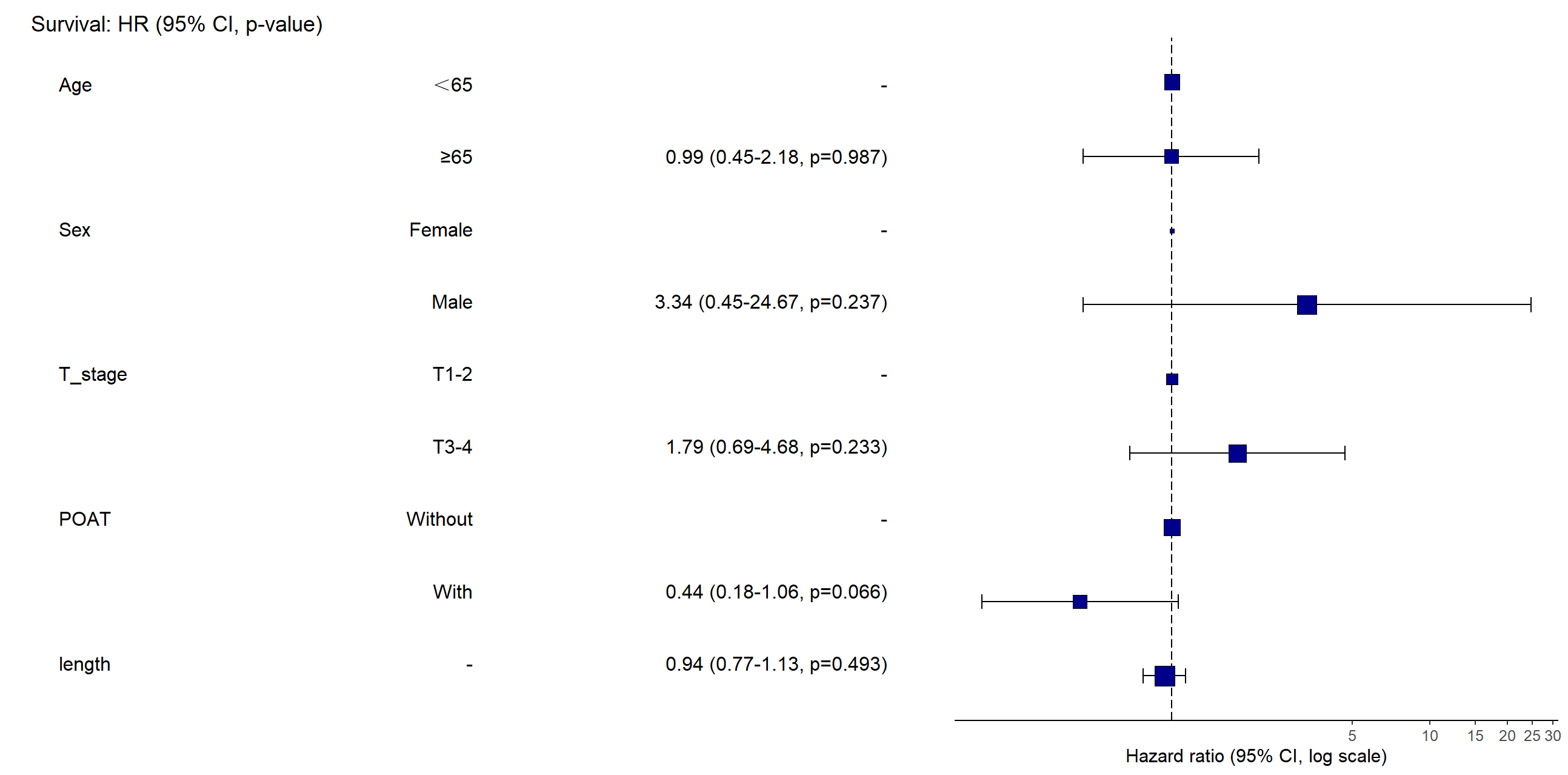


High-risk pN0 with insufficient lymph node dissection at FJCH and HNC：

Univariate Cox Regression Analysis


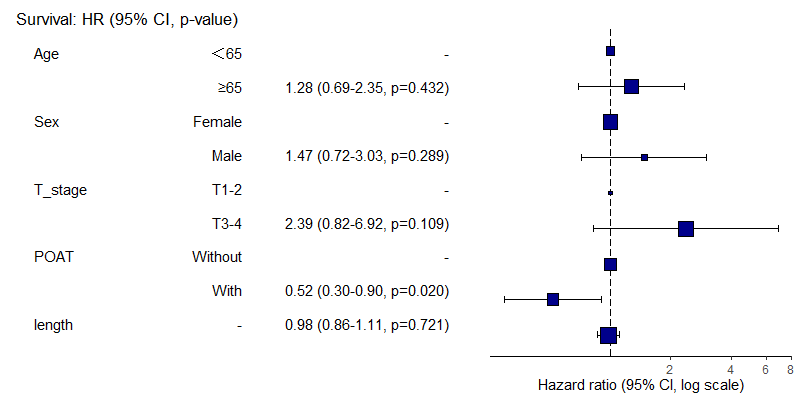


Multivariate Cox Regression Analysis


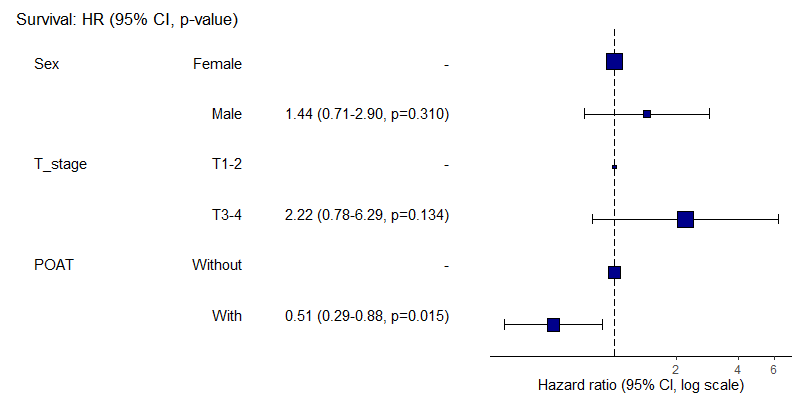


**MIL models**

The architecture of this multi-instance learning model is crafted for detailed analysis of lymph node imaging, incorporating various layers such as an embedding layer, attention mechanism, weighted sum, and classification. Here is a refined and concise description of its operation:


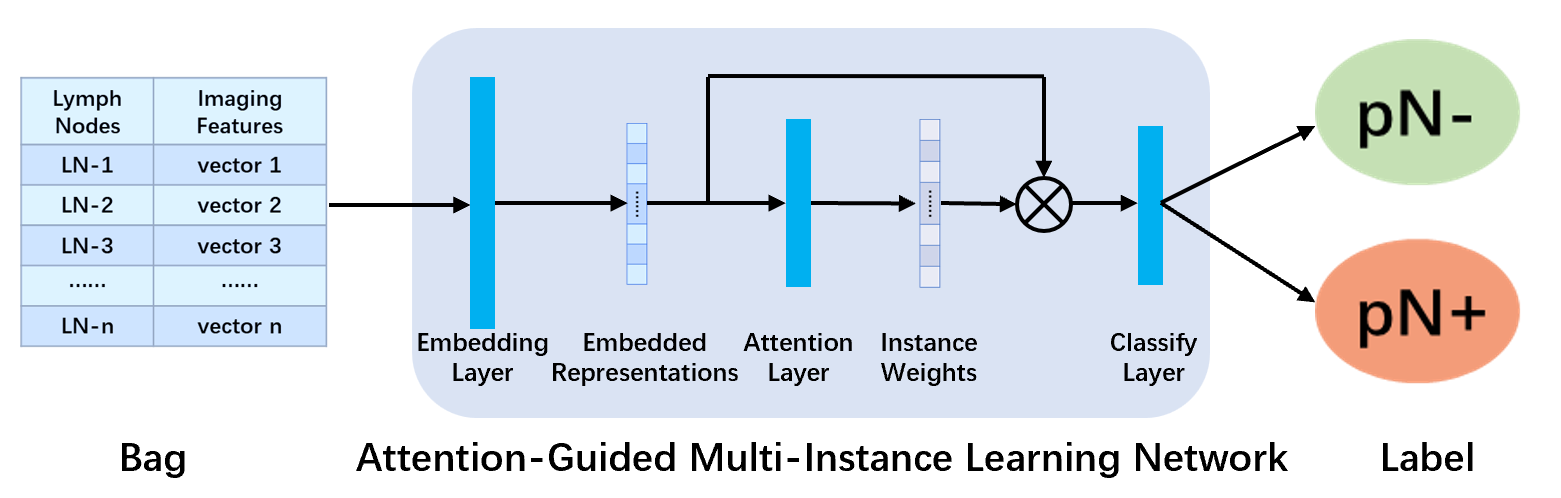


Embedding Layer: This layer utilizes a sequential network comprising fully connected layers, ReLU activation functions, and Dropout layers to perform dimensionality reduction and feature weighting on the input lymph node image features. This processing step transforms the raw lymph node features $x_{i}$, initially 512-dimensional, into a refined 32-dimensional embedded representation. The mathematical transformation from $x_{i}$ to embedding features can be expressed as:

$$e_{i}=ReLu(Dropout(W_{2}\cdot ReLu(W_{1}\cdot x_{i}+b_{1})+b_{2}))$$

Where $W_{1}\in\mathbb{R}^{128\times512}$ and $W_{2}\in\mathbb{R}^{32\times128}$ are weight matrices, and $b_{1}\in\mathbb{R}^{128}$ and $b_{2}\in\mathbb{R}^{32}$ are bias terms.

Attention Layer: This layer employs a Tanh activation function within the attention mechanism to condense the 32-dimensional embedded features into a single-dimensional score, enhancing the model's focus on significant features for prediction. The attention scores are normalized using a softmax function to derive weighted importance for each lymph node instance, facilitating targeted analysis and prediction:

$$a_{i}=W_{4}\cdot\mathrm{Tanh}(W_{3}\cdot e_{i}+b_{3})+b_{4}$$

Where $W_{3}\in\mathbb{R}^{8\times32}$ and $W_{4}\in\mathbb{R}^{1\times8}$ are weight matrices, and $b_{3}\in\mathbb{R}^{8}$ and $b_{4}\mathbb{\in R}$ are bias terms.

The normalized attention weights are calculated as:

$$\alpha_{i}=\frac{\exp(a_{i})}{\sum_{j=1}^{k} \exp(a_{j})}$$

(3) Weighted Sum: The model integrates attention weights with corresponding instance features through a weighted sum operation to produce a global representation that encapsulates information from all lymph node instances. This global feature, which prioritizes instances of significant predictive value, is computed as:

$$g=\sum_{i=1}^{k} \alpha_{i}\cdot e_{i}$$

where $\alpha_{i}$ represents the attention weight and $e_{i}$ the embedding feature vector of each instance.

(4) Classification Layer: In the final stage of the model, a classification layer comprising a fully connected layer and a ReLU activation function transforms the weighted global representation into the final prediction. This output predicts the probability 𝑦 of lymph node metastasis:

$$y=ReLu(W_{6}\cdot ReLu(W_{5}\cdot g+b_{5})+b_{6})$$

Here, $W_{5}\in\mathbb{R}^{8\times32}$ and $W_{6}\in\mathbb{R}^{2\times8}$ are weight matrices, and $b_{5}\in\mathbb{R}^{8}$ and $b_{6}\in\mathbb{R}^{2}$ are bias terms.

In the training of the model, backpropagation is utilized to update the model parameters, and to prevent overfitting while conducting feature selection, L1 regularization is incorporated:

$${L1}_{reg}=\sum_{W\in\mathrm{parameters}} \left\| W \right\|_{1}$$

where $\left\| W \right\|_{1}$ denotes the L1 norm of the weight matrix 𝑊 (i.e., the sum of the absolute values of the weights).

The overall loss function combines binary cross-entropy loss with L1 regularization:

$$Loss=-\frac{1}{N}\sum_{i=1}^{N} [y_{i}^{'}\cdot\log(y_{i})+(1-y_{i}^{'})\cdot\log(1-y_{i})]+\lambda\cdot{L1}_{reg}$$

Here, $y_{i}$ represents the predicted output of the model for the i-th patient, $y_{i}^{'}$ is the actual label for the i-th patient, and 𝜆 is the regularization coefficient. In this context, the model trained on radiomics features is named MILRad, while the model trained on deep learning features is referred to as MILDL.
